# Natural killer cell distribution and trafficking in human tissues

**DOI:** 10.3389/fimmu.2012.00347

**Published:** 2012-11-29

**Authors:** Paolo Carrega, Guido Ferlazzo

**Affiliations:** ^1^Istituto Giannina GasliniGenova, Italy; ^2^Laboratory of Immunology and Biotherapy, Department of Human Pathology, University of MessinaMessina, Italy

**Keywords:** natural killer cells, humans, chemokine receptors, cell migration, tissue distribution

## Abstract

Few data are available regarding the recirculation of natural killer (NK) cells among human organs. Earlier studies have been often impaired by the use of markers then proved to be either not sufficiently specific for NK cells (e.g., CD57, CD56) or expressed only by subsets of NK cells (e.g., CD16). At the present, available data confirmed that human NK cells populate blood, lymphoid organs, lung, liver, uterus (during pregnancy), and gut. Several studies showed that NK cell homing appears to be subset-specific, as secondary lymphoid organs and probably several solid tissues are preferentially inhabited by CD56^bright^CD16^neg/dull^ non-cytotoxic NK cells. Similar studies performed in the mouse model showed that lymph node and bone marrow are preferentially populated by CD11b^dull^ NK cells while blood, spleen, and lung by CD27^dull^ NK cells. Therefore, an important topic to be addressed in the human system is the contribution of factors that regulate NK cell tissue homing and egress, such as chemotactic receptors or homeostatic mechanisms. Here, we review the current knowledge on NK cell distribution in peripheral tissues and, based on recent acquisitions, we propose our view regarding the recirculation of NK cells in the human body.

## INTRODUCTION

Natural killer (NK) cells are a class of lymphocytes characterized by a wide spectrum of effector functions spanning from killing of target cells, mainly tumor and virally-infected cells, to the ability to influence various steps of the immune response, such as editing dendritic cells (DCs) and influencing emerging T helper response (Moretta et al., 2003; Ferlazzo, 2005; Fink et al., 2007; Vivier et al., 2011; Morandi et al., 2012). It is now clear that NK cells are not exclusively found in peripheral blood (PB) but populate different tissues and organs. Nonetheless, the true distribution of NK cells across the human body is less than clear. The task of depicting a detailed analysis of NK cell distribution is also complicated by the recent discovery of other innate populations of lymphocytes that share some characteristics (i.e., NKp46, CD56) with conventional NK cells and can populate the same compartments (Spits and Di Santo, 2011).

Contrarily to B and T cells, recirculation and trafficking of NK cells are almost unknown. Although NK cells express an ample array of chemotactic receptors, the role of the different chemokine in guiding *in vivo* trafficking of NK cells through the tissues remains poorly dissected. The distribution of NK cells seems to be subset-specific in mouse, as different NK cell subsets showed organ-specific localizations. Conversely, this issue has been poorly investigated in the human system. Here, we provide our viewpoint about distribution and trafficking of NK cells across tissues in the human system. At the same time, we will comment previous results in the light of new discoveries on NK cell specific features and on new NK-like cell populations.

## DISTRIBUTION OF NK CELLS IN HUMAN NON-LYMPHOID ORGANS

Information on NK cell distribution across human tissues was limited by methodological shortcomings, as earlier analyses often relied on the use of erroneous markers for the detection of NK cells [i.e., the use of markers then proved to be either not sufficiently specific for NK cells (e.g., CD57, CD56) or expressed only by subsets of NK cells (e.g., CD16)]. Furthermore, interpretation of studies performed with NK cells in the tissues is complicated by the fact that most studies have not been able to distinguish bona fide NK cells from the growing population of innate lymphoid cells (ILCs). In fact, as cited before, one of the challenge in identifying tissue NK cells lies in the difficulty in discriminating these cells from other NKp46^+^ ILC, such as NKp46^+^ RORγt^+^ cells. These latter ILCs, both in human and mouse, are functionally distinct from conventional NK cells and have been referred to as NK-22 cells or ILC22 cells. They share with NK cells the expression of the natural cytotoxicity receptor NKp46 and with lymphoid tissue-inducer (LTi) cells (another subset of ILCs) the expression of retinoic acid receptor-related orphan receptor-γt (RORγt). Differently from conventional NK cells, NKp46^+^ RORγt^+^ ILCs produce IL-22 and are non-cytotoxic. These NKp46^+^ RORγt^+^ ILCs are abundantly represented in mucosal tissues, particularly of the gut and oropharynx (Spits and Di Santo, 2011).

Finally, much of the studies performed to investigate human tissue NK cells were not technically able to distinguish between the two main NK cell subsets, i.e., CD56^bright^/CD16^low/neg^/KIR^neg^ NK cells (non-cytotoxic) and CD56^dim^/CD16^+^ NK cells (cytotoxic). As a result, a lack of information about relative distribution of CD56^bright^ and CD56^dim^ NK cells still remains.

Nonetheless, based on the available evidences, it seems that NK cells are present in healthy skin and gut, in the liver, in the lungs, and uterus during pregnancy. In addition, human NK cells were investigated also in other tissues such as the kidney (Schleypen et al., 2006), joints (Dalbeth et al., 2004), and breast (Faget et al., 2011) under pathophysiological conditions. In the normal intestinal mucosae, NK cells (Cella et al., 2009; Reynders et al., 2011) are found predominantly as intraepithelial lymphocytes and within the lamina propria, but are rarely associated to lymphoid aggregates, although they can be found in the parafollicular region of cecal lymphoid patches, Peyer’s patches, and mesenteric lymph nodes (LN, Chinen et al., 2007; Colonna, 2009; Halama et al., 2011). Detailed analysis of NKp46^+^ cell population showed that NKp46^+^ RORγt^neg^ cells found in the lamina propria, similarly to CD56^bright^ cells in the blood, express very low levels of perforin, if any, and contain few granzymes, suggesting that a high percentage of CD56^bright^ cells localizes in the gut (Chinen et al., 2007; Cella et al., 2009). Besides mucosae, NK cells seem to be located also in the normal skin, since it has been reported that few scattered CD56^+^ CD3^-^ cells are present in the dermis, close to the epidermis, in skin biopsy specimens from healthy individuals and in non-lesional skin from atopic eczema/dermatitis syndrome (AEDS) patients (Buentke et al., 2002). In the liver, NK cells are comprised among the non-parenchymal cells that populate this organ. In steady-state, NK cells are preferentially located in the hepatic sinusoids, often adhering to the endothelial cells (Krueger et al., 2011) and NK cells in healthy human liver strikingly account for almost 20–30% of all human hepatic lymphocytes (Doherty and O’Farrelly, 2000). Interestingly, the finding that some liver NK cells express NKp44 (Burt et al., 2009) raises the question whether this subpopulation (or at least part of it) may represent activated NK cells or the IL-22-producing NKp46^+^ RORγt^+^ cells, so far described in the intestine and tonsil. In animals, among non-lymphoid tissues, the lung contains the largest number of NK cells (Reynolds et al., 1981; Stein-Streilein et al., 1983; Basse et al., 1992; Gregoire et al., 2007; Huntington et al., 2007a). Previous studies located NK cells in the vascular and the interstitial compartments, with a smaller proportion within the airspaces and therefore accessible by bronchoalveolar lavage (Weissler et al., 1987; Basse et al., 1992). However, data available on human lung NK cells are limited and we recently revisited this topic and studied the presence and localization of NK cells infiltrating the lung tissues either normal or neoplastic. Our analyses showed that NKp46^+^ NK cells actually populate the normal lung, counting for ~10% of lymphocytes present in this tissue. In addition, we observed that the majority (~80%) of lung-NK cells belong to the CD56^dim^CD16^+^ subset (Carrega et al., 2008). Similar results were recently reported in mice, where the CD11b^high^CD27^dull^ subset (the murine subset that more similarly resembles the human CD56^dim^CD16^+^ subset) of NK cells was found to constitute approximately 90% of the lung NK cell population (Hayakawa and Smyth, 2006; Huntington et al., 2007b). A particular subset of NK cells is found in the placenta, where it regulates specific developmental processes at the fetal–maternal interface. During the first trimester of pregnancy, NK cells represent a subpopulation with unique phenotypic and functional properties, representing as much as 50–90% of the lymphoid cells infiltrating in this tissue ( King et al., 1991; Bulmer et al., 2010). Although they are characterized by a CD56^bright^CD16^neg^ phenotype, they differ from their blood counterpart in their expression of inhibitory receptors and the presence of high levels of lytic granules. Surprisingly, uterine NK cell rather than act as killers and/or drivers of inflammation, contribute to tissue building and remodeling and formation of new vessels by releasing high amounts of IL-8, VEGF, SCF-1-1, and IP-10 but low levels of IFNγ (Vacca et al., 2011). The presence of NK cells in the major human organs raises questions about how NK cells reach these peripheral organs.

## FACTORS THAT REGULATE HOMING AND TRAFFICKING OF HUMAN NK CELLS TO THE TISSUES

The expression of chemokines by organ-specific cell types suggests that organ-intrinsic elements may be important in guiding NK cell homing during physiological and pathological conditions. Differences between the main human NK cell subsets include also the expression of chemokine receptors, as CD56^dim^ and CD56^bright^ NK cell subsets largely differ in their repertoires (Campbell et al., 2001). However, besides chemokine receptors, it has been demonstrated that NK cells can additionally migrate in response to factors that do not belong to the chemokine superfamily. This is the case of the proinflammatory protein chemerin and the sphingosine 1-phosphate (S1P) molecule that can both affect trafficking of NK cells during inflammation or steady-state conditions, respectively (Parolini et al., 2007; Walzer et al., 2007). On the contrary, the chemotactic receptors expressed by NKp46 ^+^ ILC22 are largely unknown with the exception of the CCR6 receptor, which seems to promote leukocyte homing to the gut mucosa (Williams, 2006; Cella et al., 2009).

Collectively, the different chemokine receptor repertoires expressed by NK cell subsets may define entirely different routes of distribution for these cells. Nevertheless, the detailed migration patterns of NK cells have not yet been sufficiently characterized.

Furthermore, the presence of NK cells in many organs raises the question whether NK cells can exit the organs, or terminally reside in the tissues. The localization of NK cells in different tissues would suggest that: (i) they could migrate to various organs, then reside in the tissue taking on their peculiar activities, perhaps getting mobilized in the case of disease; alternatively, (ii) NK cells could re-circulate constantly through the tissues. This last hypothesis is supported by the finding that, when transferred into a naïve syngeneic host, spleen-derived murine NK cells were found in all the organs where NK cells localize and at the same proportions as host populations (Gregoire et al., 2007), thus suggesting that NK cells from one anatomical location are not restricted to that environment and can re-circulate between organs. Up to now, it is still unclear which of these alternatives might be more realistic. Based on the available data, in human, it is likely that a combination of both scenarios could be possible. As described above for the gut, liver, and lung, different human solid tissues appear to be populated by different NK cell subsets (mainly by CD56^bright^ NK cells with some exception) thus indicating that specific homing signals are important to drive localization of NK cells to the different tissues. However, the finding that human afferent lymph draining peripheral tissues contains a substantial number of NK cells (Montalto et al., 2010; and P. Carrega, personal observation) indicates that NK cells might even exit the organ and traffic through tissues in normal conditions.

## “*IN VIVO*” TRAFFICKING OF NATURAL KILLER CELLS THROUGH SECONDARY LYMPHOID ORGANS

Recent studies have demonstrated that a substantial number of NK cells are present in resting human LN and the large majority of these LN-NK cells are CD56^bright^ (Fehniger et al., 2003; Ferlazzo et al., 2004b; Vossen et al., 2008; Ferlazzo and Munz, 2009), as might be predicted by their expression of CCR7 and L-selectin (CD62L), which binds with high efficiency to physiologic L-selectin ligands on peripheral LN high endothelial venules (HEVs). Interestingly, HEVs may not represent the only route for NK cell entrance in the LN as we recently observed that NK cells are contained in afferent lymph accumulations following LN resection upon breast cancer surgery (Montalto et al., 2010; and P. Carrega, personal observations). This observation is supported by the findings that CD56^+^ lymphocytes were actually detected in human lymph draining normal skin (Hunger et al., 1999). It has also been proposed that LN may be sites for NK cell maturation. Like the bone marrow, LN can support the differentiation of NK cells from NK cell precursors through sequential maturation stages (Freud et al., 2005). Whatever their origin (developed “*in situ*” or migrated through blood and afferent lymph), NK cells are present in the T cell areas of human normal donor LN (Ferlazzo et al., 2004a,b), indicating LN as potential sites for DC/NK cell crosstalk (Ferlazzo et al., 2004b). Moreover, by performing real-time observations, it has been shown that more than 90% of the NK cells in the T cell area were found to unambiguously remain in contact with the DCs for at least 25 min (Bajenoff et al., 2006a).

Recently, sequential events regulating the egress of NK cells from secondary lymphoid organs (SLO) have been described in the mouse model. Changes in responsiveness of the S1P5 receptor to its ligand (S1P, which concentration is high in peripheral fluids) seems to have a key role in allowing NK cell exit via lymphatics (Mayol et al., 2011). However, whether this mechanism might also be effective in human has not yet been confirmed. Notably, the phenotype of NK cells exiting from LN by efferent lymph appears slightly different from that of NK cells found within these SLO. In particular, part of these cells express significant amounts of KIR and CD16, implying that CD56^bright^ NK cells could acquire these molecules in the LN during inflammation and then circulate through the efferent lymph into PB as KIR^+^CD16^+^ NK cells (Romagnani et al., 2007). This notion is also supported by the finding that NK cells of highly reactive LN partially express KIRs and CD16 (Romagnani et al., 2007). Nevertheless, it cannot be excluded that CD16^+^KIR^+^ NK cells might reach LN because of local inflammation, as better discussed below.

## ARE INFLAMED PERIPHERAL TISSUES SITES FOR NK CELL MATURATION?

**FIGURE 1:**
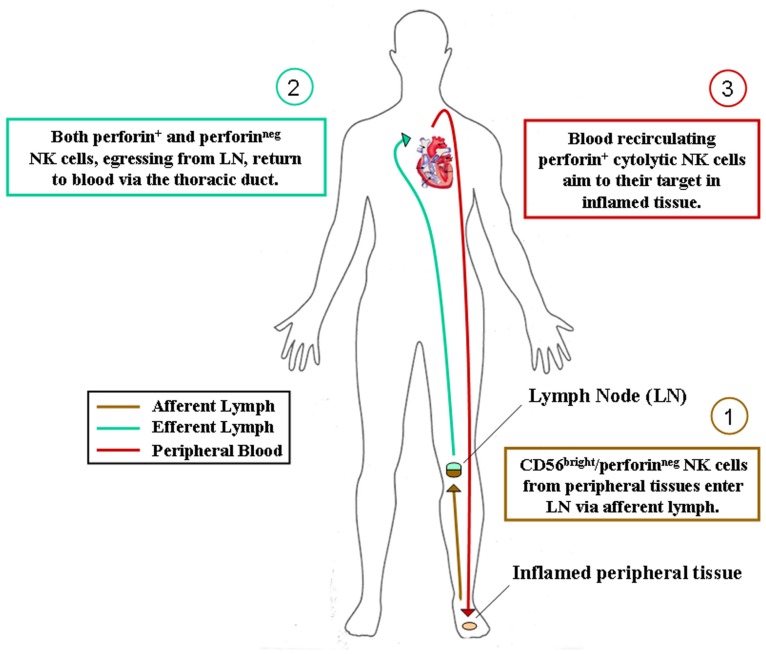
**Hypothesis of NK cell subset recirculation in human body**. After their generation in the bone marrow, CD56^bright^/perforin^neg^ NK cells colonize most human tissues according to their chemokine receptor repertoire. They can eventually acquire cytolytic functions during immune response in inflamed tissues and act locally as innate effector cells. Alternatively, as depicted, they can reach secondary lymphoid organs via afferent lymph, acquire perforins and cytolytic functions during immune reactions occurring in lymphoid organs and then recirculate in peripheral blood for exerting their protective activities in inflamed tissues.

It has been demonstrated that, upon activation, CD56^bright^ NK cells could acquire the signature of CD56^dim^ NK cells, i.e., perforin^+^, KIR^+^, CD16^+^, IL-7R^neg^, c-*kit*^neg^, CXCR3^neg^, CCR7^neg^, CD62L^neg^, whereas CD56^dim^ CD16^+^KIR^+^ NK cells substantially maintain their features of terminally differentiated cells (Romagnani et al., 2007). Even if this process was elegantly demonstrated *in vitro*, the sites and physiological conditions where this process may happen remain almost unknown *in vivo*. Nevertheless, several observations seem to support the hypothesis that similar steps of NK cell differentiation occur also *in vivo*. Indeed, it has been observed that non-reactive LN contain almost exclusively CD56^bright^ KIR^neg^ CD16^neg^ NK cells, whereas a significant expression of KIR and CD16 is present in NK cells contained in highly inflamed LN and in the efferent lymph. Thus, it could be envisaged that in steady state or very early during an immune response, CD56^bright^ KIR^neg^ NK cells can be recruited into LN (Martin-Fontecha et al., 2004; Bajenoff et al., 2006b), whereas later on during inflammation mature NK cells leave LN and then circulate in PB to reach inflamed tissues (**Figure 1**). Although this hypothesis is very challenging, it cannot be excluded that the presence of KIR^+^CD16^+^ NK cells in inflamed LN (and in the efferent lymph) might be due to selective migration of this subset into LN. This latter theory is supported by some *in vitro* data, which showed that stimulation of human PB CD56^dim^ cells NK cells with IL-18 can up-regulate their expression of CCR7 (Mailliard et al., 2005). However, data derived from the investigation of tumor-infiltrating NK cells, support the hypothesis that, also *in vivo*, local activation could convert CD56^bright^CD16^neg^ NK cells into effectors analogous to blood CD56^dim^CD16^+^ NK cells. Our observations in NK cells infiltrating lung cancers suggest that up-regulation of KIR on CD56^bright^CD16^neg^ perforin^neg^ NK cells can occur also *in vivo*, most likely because of a proinflammatory cytokine microenvironment due to local immune reactions at the tumor site (Carrega et al., 2008; and P. Carrega, unpublished observations). Therefore, we should consider that, besides normal distribution and homeostatic re-circulation, the presence and functions of NK cells in a specific tissue could also be influenced by *in situ* differentiation due to local microenvironmental factors.

## CONCLUDING REMARKS

Recent studies have significantly increased our knowledge on NK cell distribution in some human peripheral tissues, such as SLO and uterus. However, information on NK cells presence in most human solid tissues, as well as on how NK cell redistribute during pathological processes, is still largely lacking. Investigations in this field are now highly required in view of novel attempts of NK cell-based adoptive immunotherapies in both cancer and organ transplantation settings.

The few earlier data on NK cells infiltrating human peripheral tissues have often been obtained using markers then proved to be either not sufficiently specific for NK cells or expressed only by subsets of NK cells. In addition, nowadays, we appreciate that, in solid tissues, classical NK cells must be distinguished from other RORγt^+^ ILCs, which, despite functionally distinct, share several conventional NK cell hallmarks. On the basis of what we learned in the last years regarding innate lymphocytes, some previous correlations between NK cell infiltration and clinical status, such as cancer prognosis, should now probably be reconsidered and further dissected.

Apart from these newly described RORγt^+^ NKp46^+^ cells, the most recent analyses on human tissue NK cells have shown that perforin^low/neg^, non-cytotoxic, NKp46^+^ cells represent in our body a population of cells much larger than previously realized. Accumulating evidences are indicating that, in addition to SLO, also many other human solid tissues, including some cancer histotypes, might predominantly host the CD56^bright^/KIR^neg^/perforin^neg^ NK cell subset rather than the cytotoxic perforin^+^ counterpart, which is conversely largely dominant in PB.

We have recently identified NKp46^+^ cells within human afferent lymph and further analyses are currently performed in order to confirm and extend these preliminary results. Nonetheless, also in consideration of previous studies on developmental relationships of NK cell subsets and on the distribution of NK cell subsets in SLO, inflamed tissues, and efferent lymph (Ferlazzo et al., 2004b; Romagnani et al., 2007), we are now prone to hypothesize that CD56^bright^ non-cytotoxic NK cells, similarly to naïve T cells, could re-circulate through afferent lymph from peripheral tissues to SLO. Here, they could achieve or not their final maturation (depending on whether or not an immune reaction will occur inside the LN) and then re-circulate via venous blood vessels or efferent lymph to PB (**Figure 1**).

Thus, reactive SLO might be the sites for NK cell final maturation, acquisition of cytotoxic properties and tolerance to self (KIR expression). Complete maturation of NK cells is indeed accompanied by the switching of the chemokine receptor repertoire, which would drive blood re-circulating cytotoxic NK cells toward their targets in inflamed pathological tissues. Alternatively, NK cells could also achieve their final differentiation steps directly in inflamed tissues, where tissue resident non-cytotoxic CD56^bright^ perforin^neg^ NK cells might differentiate into perforin^+^ final effector NK cells during the local immune response against invading pathogens.

## Conflict of Interest Statement

The authors declare that the research was conducted in the absence of any commercial or financial relationships that could be construed as a potential conflict of interest.

## Acknowledgments

Research in our laboratory is supported by Associazione Italiana Ricerca sul Cancro (AIRC) IG11650 to Guido Ferlazzo; Paolo Carrega is a recipient of a postdoctoral fellowship from the Fondazione Italiana per la Ricerca sul Cancro (FIRC).
